# Annotated genome of *Aedes japonicus japonicus* using a hybrid-assembly approach

**DOI:** 10.3389/fgene.2025.1667262

**Published:** 2025-10-01

**Authors:** Friederike Reuss, Tilman Schell, Haruhiko Isawa, Shinji Kasai, Sven Klimpel, Ruth Müller, Markus Pfenninger, Judith Kochmann

**Affiliations:** ^1^ Institute of Occupational, Social and Environmental Medicine, Goethe University Frankfurt, Frankfurt am Main, Germany; ^2^ Senckenberg Research Institute, Frankfurt am Main, Germany; ^3^ LOEWE Centre for Translational Biodiversity Genomics (LOEWE-TBG), Frankfurt am Main, Germany; ^4^ Department of Medical Entomology, National Institute of Infectious Diseases, Japan Institute for Health Security (JIHS), Tokyo, Japan; ^5^ Institute for Ecology, Evolution and Diversity, Goethe-University, Frankfurt am Main, Germany; ^6^ Senckenberg Biodiversity and Climate Research Centre (SBiK-F), Frankfurt am Main, Germany; ^7^ Unit of Entomology, Institute of Tropical Medicine Antwerp, Antwerp, Belgium; ^8^ Institute of Organismic and Molecular Evolution (iomE), Johannes Gutenberg University, Mainz, Germany

**Keywords:** complete mitochondrial sequence, *Aedes*, invasive mosquitoes, disease vector, reference genome

## 1 Introduction

Mosquitoes (Diptera: Culicidae) are an important group of insects due to the important role played by culicid species as disease vectors. Some *Aedes* species are competent to vector human and veterinary relevant viruses, such as dengue, chikungunya, or Japanese encephalitis viruses. In addition, there are some highly invasive *Aedes* species (Lounibos, 2002). The two most widespread species globally are *Aedes albopictus*, native to Southeast Asia, and *Aedes aegypti*, native to Africa, for which genomes have been sequenced previously: *Ae. aegypti* AaegL5.0 (GCF_002204515.2; Matthews et al., 2018) and *Ae. albopictus* AalbF5 (GCF_035046485.1; Palatini et al., 2020). Globally, *Ae. aegypti* is the primary vector of chikungunya and dengue viruses (Sousa et al., 2012; Jansen et al., 2018). *Aedes albopictus* is a secondary vector to *Ae. aegypti* for chikungunya and dengue viruses (Jansen and Beebe, 2010; Sousa et al., 2012); however, it is the most important vector for autochthonous cases of dengue and chikungunya in Europe (Rezza et al., 2007; Gjenero-Margan et al., 2011; Succo et al., 2016). Both *Ae. aegypti* and *Ae. albopictus* are invasive species in Europe (European Centre for Disease Prevention and Control and European Food Safety Authority, 2023).

Another more recent invader to North America (Kaufman and Fonseca, 2014) and Europe is *Aedes japonicus japonicus*, while its sister species *Aedes koreicus* has established itself in Europe (European Centre for Disease Prevention and Control and European Food Safety Authority, 2023). Over the last two to three decades, *Ae. j. japonicus* has spread beyond its original area of distribution in East Asia via the import of used tires and trade (Kaufman and Fonseca, 2014; Koban et al., 2019) and is likely to expand its range of area distribution in the future (Cunze et al., 2020). Annotated genomes for *Ae. j. japonicus* and *Ae. koreicus* (GCA_034211315.2, GCA_024533555.2) have only recently become available (Catapano et al., 2023; Nagy et al., 2024).

Here, we describe an annotated genome and a complete mitochondrial sequence of *Ae. j. japonicus* from a laboratory strain in Japan (Hoshino et al., 2010). This is the first study wherein individuals from the native range of this species (Kaufman and Fonseca, 2014) were sequenced.

The mitochondrion of *Ae. j. japonicus* can help in constructing phylogenies. For example, the genus *Aedes* and the tribe of Aedini have been re-organized based on morphological analyses (reviewed in Wilkerson et al., 2015) and molecular analyses (Zadra et al., 2021). Thus, genetic datasets are highly desirable for creating a well-founded phylogeny of Aedini or *Aedes* (Zadra et al., 2021).

Our genome assembly can facilitate marker selection for environmental associations and genotype-to-phenotype-association studies. By doing so, the genomic basis of vector competence or invasion success can be identified within the species *Ae .j. japonicus* and also compared to that of other *Aedes* spp. More specifically, the created dataset allows conducting comparative studies regarding diapause (Kreß et al., 2016; Boyle et al., 2021), thermotolerance (Kramer et al., 2023; Couper et al., 2025), and population structure (Smitz et al., 2021), all considered potential parameters influencing invasiveness (Lahondère and Bonizzoni, 2022).

Although *Ae. albopictus* and *Ae. aegypti* are the primary vectors of dengue and chikungunya viruses, *Ae. j. japonicus* is only a minor vector in the transmission of disease agents, and its vector competence is largely based on laboratory competence studies (Medlock et al., 2012; Jansen et al., 2018; Wagner et al., 2018). Both *Ae. j. japonicus* and *Ae. albopictus* can undergo photoperiodic diapause (Armbruster, 2016; Krupa et al., 2021), which benefits the species’ survival in more temperate regions. In addition, this dataset provides data to study candidate genes related to not only vector competence but also insecticide resistance. It also provides genomic resources for marker identification, which can be used in eDNA approaches for a more rapid species detection in the field (Wittwer et al., 2024), genetic control measures such as gene drives, *Wolbachia*-based methods (Verkuijl et al., 2025; Wang et al., 2025), or RNA interference (Müller et al., 2023).

## 2 Methods

### 2.1 Origin of biological material and DNA isolation

**FIGURE 1:**
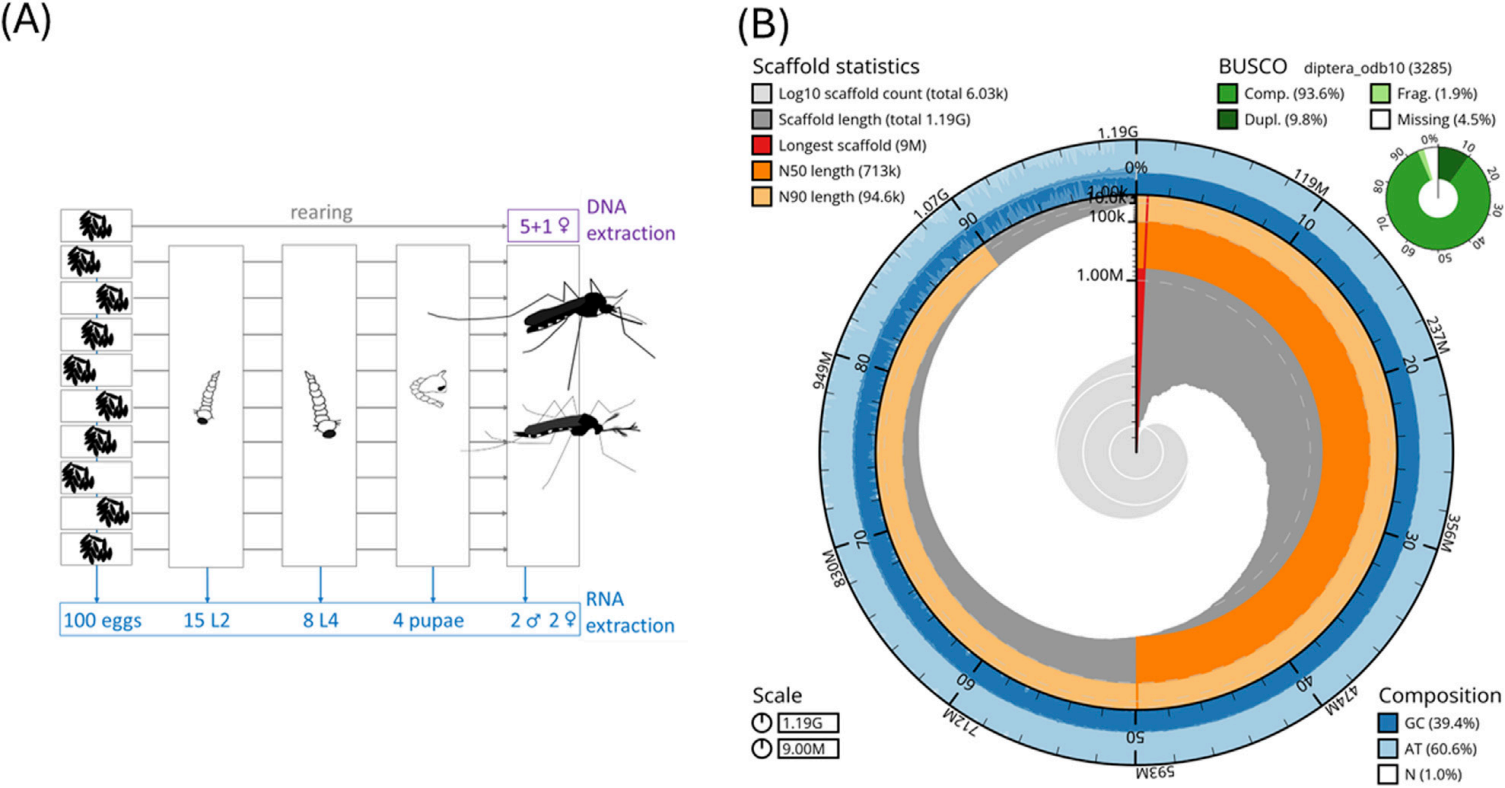
**(A)** Biological material for DNA and RNA isolation. We used closely related (offspring of one female) individuals for DNA isolation to minimize variation. **(B)** Snail plot of statistics of the *Ae. j. japonicus* assembly.

For DNA and RNA isolation, the offspring of ten female *Ae. j. japonicus* were collected during the egg stage from the “Narita” laboratory strain (Hoshino et al., 2010) and raised to the desired stages (Figure 1A) for DNA and RNA isolation.

A pool of five sister species in the adult stage was used for DNA MinION long-read and Illumina short-read sequencing, while a single adult female (another sister) was used for PacBio DNA sequencing. DNA was isolated using the protocol “HMW gDNA Extraction from Single Insects” (10x Genomics, Pleasanton, CA, United States). The fragment size distributions and DNA concentrations were assessed using TapeStation (Agilent Technologies, Santa Clara, CA, United States) and Qubit Fluorometer measurements using the DNA BR kit (Thermo Fisher Scientific, Waltham, MA, United States).

### 2.2 DNA sequencing data

The Illumina sequencing provider (BGI Hong Kong) handed over already filtered, so-called clean reads in eight pairs. These paired-end read files were adapter-trimmed using autotrim 0.6.1 (Waldvogel et al., 2018) and its dependencies FastQC, Trimmomatic 0.39 (Bolger et al., 2014), and MultiQC (Ewels et al., 2016). After a quality-check, one file pair was additionally cropped to 140 bp in length using Trimmomatic 0.39. All trimmed reads were combined into one forward, one reverse (both paired-end), and one unpaired fastq file. Illumina reads were classified in Kraken 2 (paired-end files with the additional option-paired) using a customized database consisting of the Kraken 2 databases “bacteria,” “archaea,” “human,” and “UniVec-Core”.

MinION library preparation followed the manufacturer’s protocol for the 1D-ligation kit (SQK-LSK109) of Oxford Nanopore Technologies (ONT). In total, eight flow cells in three runs were used. ONT-basecalling from fast5 files was conducted with Guppy 3.4.5 (available via registering at https://nanoporetech.com/support) using default settings and the following specifications: the flowcell ID, the name of the kit used for library preparation (SQK-LSK109), and the device (device auto). For the single female species, one run on the PacBio Sequel II in CCS mode was performed. The Guppy-basecalling includes adapter trimming and Q-score-filtering.

### 2.3 RNA sequencing

For RNA extractions, 100 eggs, 15 L2 larvae, eight L4 larvae, four pupae, and two adult male and two adult female species were used (Figure 1A). Tissue samples were collected in TRIzol and extracted using the Zymo RNA Kit (Zymo Research). Eggs, larvae, and pupae were pooled for producing an immature pool. The fragment size distributions and RNA concentrations per pool were assessed using TapeStation (Agilent Technologies) and a Qubit Fluorometer with the Qubit RNA HS kit measurements (Thermo Fisher Scientific). Library construction and sequencing on a BGISEQ-500 Illumina platform were carried out at BGI Hong Kong. Raw RNA Illumina reads were quality-checked and adapter-trimmed using autotrim 0.6.1 (Waldvogel et al., 2018) and its dependencies FastQC, Trimmomatic 0.39 (Bolger et al., 2014), and MultiQC (Ewels et al., 2016). HISAT2 (Kim et al., 2019) was used to map the RNA sequencing reads to the genome assembly.

### 2.4 Mitochondrial genome

Raw PacBio circular consensus sequencing (CCS) reads with adapters were used in NOVOPlasty 4.2 (Dierckxsens et al., 2016) to assemble the mitochondrion of *Ae. j. japonicus*. For annotations, GeSeq (Tillich et al., 2017) and MITOS2 Galaxy 2.0.6 (Al Arab et al., 2017; Donath et al., 2019) were used. Using Geneious Prime 2021.2.2 (Biomatters Limited), the origin was manually set, the sequence was circularized, and the annotations were curated manually.

### 2.5 Genome size estimations

We used two *in silico* genome size estimation methods based on k-mers and read mapping. Jellyfish 2.3.0 (Marçais and Kingsford, 2011) was used to count k-mers in the *Ae. j. japonicus* Illumina paired-end reads processed by Kraken 2 v2.0.8 (Wood et al., 2019), which were returned as unclassified. The online version of GenomeScope 2.0 (Ranallo-Benavidez et al., 2020) was used to estimate a k-mer-based genome size (Supplementary Figure S1). backmap.pl v0.5 (Schell et al., 2017; Pfenninger et al., 2022) (dependencies: bwa 0.7.17-r1188, minimap 2 2.29-r1283, samtools 1.20, qualimap 2.2.1, bedtools 2.28.0, and multiqc 1.9) was used to estimate the fraction of the assembled reads via the mapping rate and for genome size estimation with the ModEst method (Pfenninger et al., 2022).

Flow cytometry was used as a sequencing-free method for genome size estimation. Genome sizes for *Ae. j. japonicus* and *Ae. koreicus* were estimated following a flow cytometry protocol with propidium iodide-stained nuclei (Hare and Johnston, 2012) using the modification of the method proposed by Männer et al. (2024). We included *Ae. koreicus* here because no flow cytometric genome size estimate exists for this species (Supplementary Table S1). One whole adult mosquito was used per suspension and chopped with a razor blade in a Petri dish. Two adults per species (one male and one female each, collected as sympatrically occurring pupae on the graveyard Wiesbaden–Kloppenheim on 27 May 2025, and lab-reared to adults) were measured on three consecutive days to minimize instrumental errors.

### 2.6 Genome assembly, scaffolding, and gap closing

A *de novo* genome was assembled with PacBio CCS reads with the Flye 2.8 assembler (Kolmogorov et al., 2019). We identified the mitochondrial sequence in the Flye assembly using blast 2.10.0 (Altschul et al., 1990), and the respective contigs (>90% target sequence identity and all blast hits per contig >70% contig length) were removed to ensure that the mitochondrion was removed but nuclear mitochondrial DNA segments (NUMTs) were retained in the nuclear genome.

Subsequently, several rounds of scaffolding and gap closing were conducted (Supplementary Figure S2): The MinION long reads were used to scaffold the Flye assembly using SLR (Luo, 2014). TGS-GapCloser 1.0.1 (Xu et al., 2020) was applied to close gaps by first using the PacBio CCS reads and then the constructed continuous long reads (“CLR” reads) together with Illumina reads. The latter were used for polishing the newly added “CLR”-gap sequence inside TGS-GapCloser. “CLR” reads are all PacBio subreads, which were not involved in the generation of a CCS read. They were filtered for the longest per zero-mode waveguide. After this sequence extension, SSPACE (Boetzer et al., 2011) was used to re-scaffold using the “CLR” reads, followed by another two-step gap closing with TGS-GapCloser using CCS reads and “CLR” and Illumina reads, as described above. This workflow allowed the incorporation of all the generated sequencing data (MinION long reads, Illumina short reads, and PacBio CCS reads) into the genome assembly (Supplementary Figure S2).

Every step of the genome assembly was evaluated regarding quality using QUAST 5.0.2 (Gurevich et al., 2013) and regarding completeness using BUSCO 5.4.6 with the diptera_odp10 gene set in the genome mode. The process of gap closing and scaffolding (Supplementary Figure S2) was checked to ensure no reduction in the quality of the resulting assembly.

### 2.7 Structural annotation

A reference-based annotation of the *Ae. j. japonicus* genome was produced using the GeMoMa 1.9 software (Keilwagen et al., 2019), own RNA sequencing data, and the *Ae. albopictus* and *Ae. aegypti* annotations for reference (GCF_035046485.1; GCF_002204515.2). The annotation of *Ae. koreicus* (GCA_024533555.2) was additionally included as a third reference in a second GeMoMa run (Supplementary Table S3).

In addition, an annotation with BRAKER 3.0.3 (Stanke et al., 2008; Li et al., 2009; Barnett et al., 2011; Lomsadze et al., 2014; Buchfink et al., 2015; Hoff et al., 2016; Brůna et al., 2021) with RNA sequencing data as evidence was computed.

BRAKER and GeMoMa annotations for *Ae. j. japonicus* were compared regarding contiguity statistics that were calculated with a custom script by author TS (named “contiguity statistics” in Table 1, Supplementary Tables S2, S3) and regarding BUSCO 5.4.6 statistics using the protein sequences as input (Supplementary Table S2). Complete and single-copy BUSCO gene IDs unique to the GeMoMa annotation were extracted and merged with the BRAKER annotation’s BUSCO IDs using gff-merge and gff3_to_fasta of the GFF3toolkit 2.1.0 (Chen et al., 2019). Since the merging did not improve the BRAKER annotation substantially (Supplementary Figure S4; Supplementary Table S2), the latter alone was used for subsequent analyses.

**TABLE 1:** Genome assembly (A) and annotation statistics (B) of selected *Aedes* spp. genomes. Calculations of contiguity statistics by a custom script. CDS: coding exon regions. Total gene space: sum of all nucleotides that are annotated as a gene. Single CDS mRNA: number of mRNAs that only have a single coding exon.

(A) Assembly statistics					
*Aedes* species	*j. japonicus* This study	*japonicus* GCA_034211315.2	*koreicus* GCA_024533555.2	*albopictus* GCF_035046485.1	*aegypti* GCF_002204515.2
Quast					
No. of scaffolds	6,029	25,235	6,100	1,497	2,310
Total scaffold length (bp)	1,185,987,502	1,389,713,034	1,100,040,858	1,344,164,507	1,278,732,104
Scaffold N50 (bp)	712,605	118,241	329,610	450,188,506	409,777,670
No. of Ns per 100 kbp	999.63	199.05	2.82	125.87	1.79
No. of contigs	6,744	25,703	6,127	6,007	2,539
Total contig length (bp)	1,174,131,623	1,386,947,059	1,100,009,795	1,342,452,197	1,278,709,169
Contig N50 (bp)	677,340	112,964	329,031	1,015,000	11,758,062
GC%	39.44	39.50	39.67	40.33	38.18
%BUSCO (n = 3,285)					
Complete	92.9	92.4	84.0	95.7	96.7
Single-copy	83.5	78.8	70.7	90.7	93.4
Duplicated	9.4	13.6	13.3	5.0	3.3
Fragmented	1.7	2.4	2.7	1.6	1.6
Missing	5.4	5.2	13.3	2.7	1.7

(B) Annotation statistics				
*Aedes* species	*j. japonicus* This study	*koreicus* GCA_024533555.2	*albopictus* GCF_035046485.1	*aegypti* GCF_002204515.2
Continuity statistics				
No. of genes	23,878	21,377	23,630	18,293
No. of mRNA	28,836	22,580	33,058	28,304
No. of CDS	120,432	87,069	171,395	173,240
Mean mRNAs/gene	1.21	1.06	1.40	1.55
Mean CDSs/mRNA	4.18	3.86	5.18	6.12
Median gene length (bp)	2,027	2,068	4,392	5,172
Median mRNA length (bp)	2,223	2,081	14,453	29,581
Median CDS length (bp)	180	217	196	187
Total gene space (bp)	407,892,850	144,700,806	738,863,314	683,632,137
Total mRNA space (bp)	407,892,850	144,698,715	702,543,701	669,443,925
Total CDS space (bp)	28,457,713	24,159,712	38,510,628	24,237,954
Single CDS mRNA (bp)	4,854	3,931	5,908	2,031
%BUSCO (n = 3,285)				
Complete	91.4	81.2	98.5	99.4
Single-copy	62.3	48.7	61.8	60.5
Duplicated	29.1	32.5	36.7	38.9
Fragmented	2.8	2.8	0.2	0.2
Missing	5.8	16.0	1.3	0.4

### 2.8 Functional annotation and detection of integrated virus sequences

InterProScan 5.61.93 (Jones et al., 2014) with the options [-f tsv -iprlookup -pa -goterms -dp -cpu 54] and blastp 2.14.0 with options [-num_threads 70 -max_hsps 1 -max_target_seqs 1 -outfmt 6] were run against the Swiss-Prot database (The UniProt Consortium et al., 2025); Pannzer2 web version (Törönen and Holm, 2022) and GhostKOALA web version (Kanehisa et al., 2016) were run to functionally annotate the amino acid file of the *Ae. j. japonicus* BRAKER annotation and the annotations of *Ae. albopictus* and *Ae. aegypti* for comparison (Supplementary Table S4; Supplementary Figure S5).

Integration of viral sequences was checked using a published database for endogenous viral elements (Palatini et al. 2020; their additional file 4) identified (tblastn 2.14.0 with options [-max_hsps 1 -max_target_seqs 1 -outfmt 6]; Altschul et al., 1990) in the respective *Aedes* amino acid files (Supplementary Table S4; Supplementary Figure S5).

## 3 Data analysis

### 3.1 Mitochondrion

The mitochondrial genome is available under the GenBank accession-number MZ566802 and NCBI accession-number NC_081591.1. The total length is 16,848 bp. As of 25 June 2025, seven additional complete mitochondrial sequences of the species are available (OP373191.1, OR668893-4.1, PQ588181.1, and PV094741-3.1), generated from mosquitoes originating from Italy, Germany, the Netherlands, and Hawaii, USA. Thus, this is the first *Ae. j. japonicus* mitochondrion from the species’ native range (Japan).

### 3.2 Assembly and genome size estimates

An *Ae. j. japonicus* assembly was obtained with a total length of 1.2 Gb, a contig N50 of 677 kb, a scaffold N50 of 712 kb, and 6,029 scaffolds (Figure 1B; Table 1A). The BUSCO protein set was 92.9% complete, with only 1.7% fragmented BUSCOs (Figure 1B). Flow cytometric genome size estimates were 1.3 Gb for *Ae. j. japonicus* as well as for *Ae. koreicus* (Supplementary Table S1). The latter is in line with the size of the *Ae. koreicus* genome (1.1 Gb; Supplementary Table S1; Nagy et al., 2024). The k-mer-based estimate of *Ae. j. japonicus* was 695 Mb in length, and the mapping-based estimate was the best performing, regarding peak shape, with mapped CCS reads. The mapping-based genome size estimate was 1.2 Gb (Supplementary Figure S3). This compilation of genome size estimates can facilitate calculations for genome coverage and sequencing costs for further projects.

### 3.3 Structural and functional annotations

The annotation with BRAKER resulted in 23,878 predicted protein-coding genes with a median length of 2,027 bp. Protein sequences of the predicted genes showed a BUSCO completeness of 91.4% (Table 1B). Among the protein-coding genes, 99% (28,458 genes) could be functionally annotated with at least one of the applied methods, but GO terms could be found for 60% of the sequences (Supplementary Table S4).

### 3.4 Comparisons to other *Aedes* genomes

The size of the nuclear genome assembly of *Ae. j. japonicus* is comparable to those of other genomes within *Aedes* (Supplementary Table S1). The *Ae. j. japonicus* assembly has slightly better statistics than the publicly available assembly (GCA_034211315.2) regarding continuity and BUSCO completeness (Table 1A). The GC content is the same as in the GCA_034211315.2 assembly and comparable to the sister species *Ae. koreicus* (Table 1A). For the three *Aedes* species, a comparable number (60%–70%) of integrated virus sequences could be detected (Supplementary Table S4; Supplementary Figure S5). The slightly lower number of viruses that could be recovered in the *Ae. japonicus* annotation is explainable by the lower quality of the scaffold-level *Ae. j. japonicus* genome compared to that of the chromosome-level genomes of *Ae. albopictus* and *Ae. aegypti* or the selection of the input virus database. A biological reason could be the species-specificity of viral integrations.

## 4 Dataset usage and availability

### 4.1 Dataset re-use potential

The dataset presented here can be used in subsequent analyses regarding phylogeny, evolution of diapause and invasiveness, adaptation to non-native habitats, and the search for genetic targets of vector control measures. It is the first time that individuals from the native range of *Ae. j. japonicus* were sequenced (nuclear and mitochondrial genomes), allowing comparative studies regarding differences between native and invasive populations of the species. Differences could occur due to the adaptation to the new environment during the invasion process. Important phenotypic traits such as diapause, heat tolerance, or insecticide resistance could be altered during invasion. The dataset presented here also fills a gap of knowledge regarding comparative studies between well-studied primary (*Ae. aegypti* and *Ae. albopictus*) and understudied secondary (*Ae. j. japonicus* and *Ae. koreicus*) vector species regarding their different competences for arboviral transmission.

## Data Availability

All datasets are available in publicly accessible repositories: This project was registered under the BioProject number PRJNA1085103 at NCBI https://www.ncbi.nlm.nih.gov/. The mitochondrial genome is available under GenBank accession-number MZ566802 https://www.ncbi.nlm.nih.gov/. The genome annotation with the corresponding assembly and the amino acid sequence file can be found at the Goethe University Data Repository (GUDe; https://gude.uni-frankfurt.de/home) under the DOI (https://doi.org/10.25716/gude.12xf-dt1*).
