# Supplementary material for: Annotated genome of Aedes japonicus japonicus using a hybrid-assembly approach
Source: Front Genet. 2025 Oct 1;16:1667262. doi: 10.3389/fgene.2025.1667262 (PMC12520638; doi:10.3389/fgene.2025.1667262)
Supplement: Supplementary file 1 [file Supplementaryfile1.docx]

Supplementary material

Authors:

Friederike Reuss, Tilman Schell, Haruhiko Isawa, Shinji Kasai, Sven Klimpel, Ruth Müller, Markus Pfenninger, Judith Kochmann

**
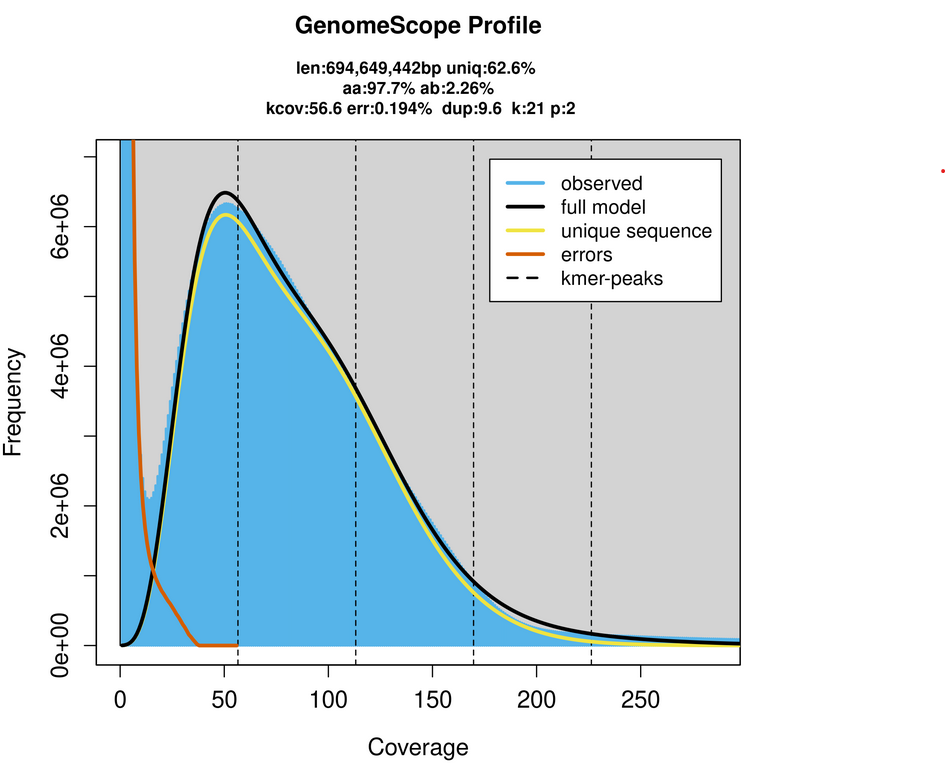
**

Supplementary figure 1: **Result of the k-mer-based genome size estimate using GenomeScope version 2.0** [2] **and Illumina reads.** The estimated genome length is about 695 Mb.


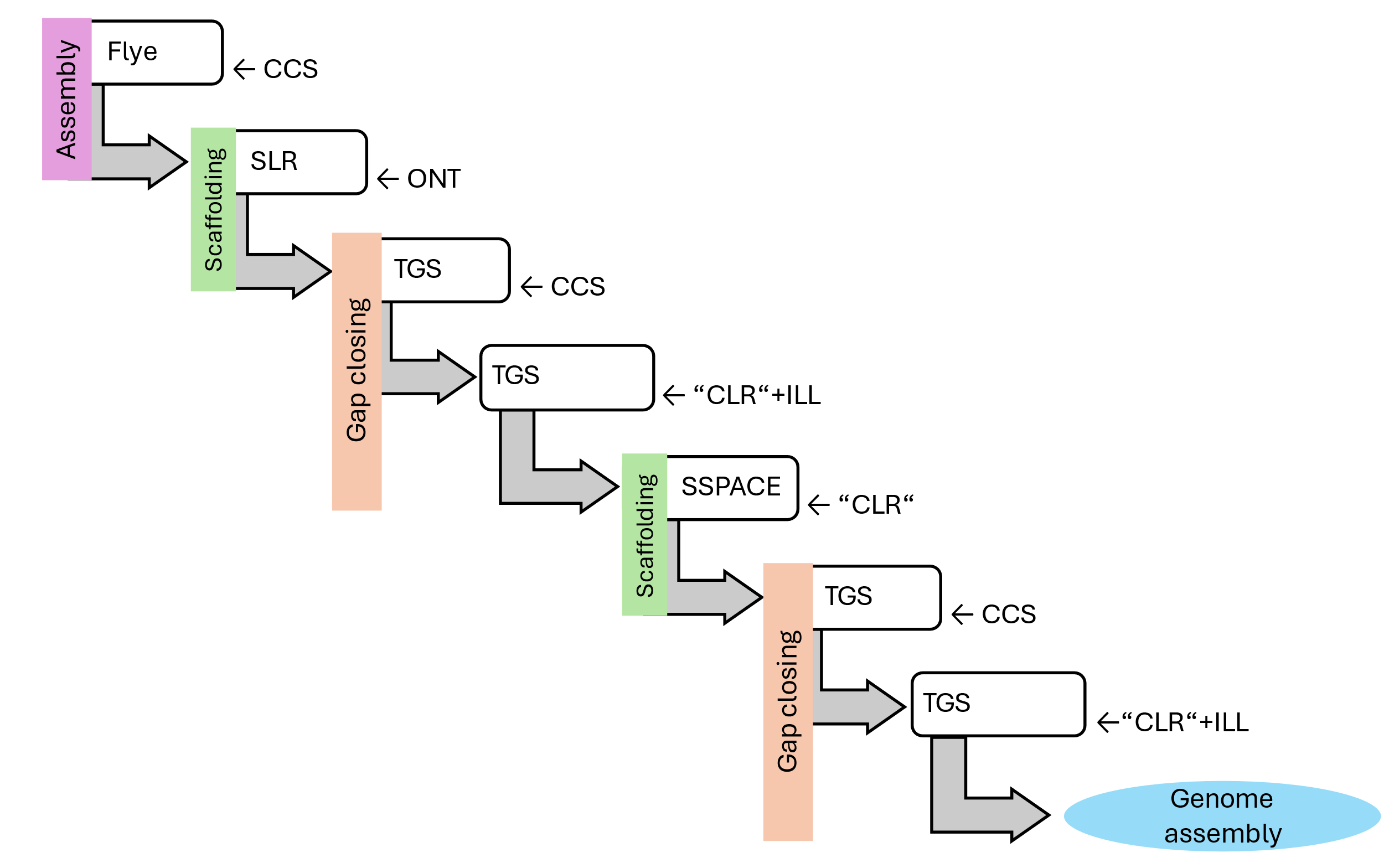


Supplementary figure 2: **Flow-chart of the gap closing-process.** CCS: raw PacBio circular consensus sequencing (CCS) reads, ONT: Oxford Nanopore Technology’s MinION long reads, TGS: TGS-GapCloser 1.0.1 (<https://github.com/BGI-Qingdao/TGS-GapCloser>), Flye: Flye assembler [1], “CLR”: all subreads, which were not involved in any generation of a CCS read, were filtered for the longest per ZMW (zero-mode waveguide). As a result, we received long PacBio reads with a higher error rate compared to CCS reads, which are overall comparable to continuous long reads (CLR) reads. ILL: Illumina reads, which were used to polish the extensions made with “CLR” reads. SSPACE: SSPACE software (<https://github.com/nsoranzo/sspace_basic/tree/main>).

Supplementary figure 3: **Coverage distributions of mappings per used sequencing technology.**
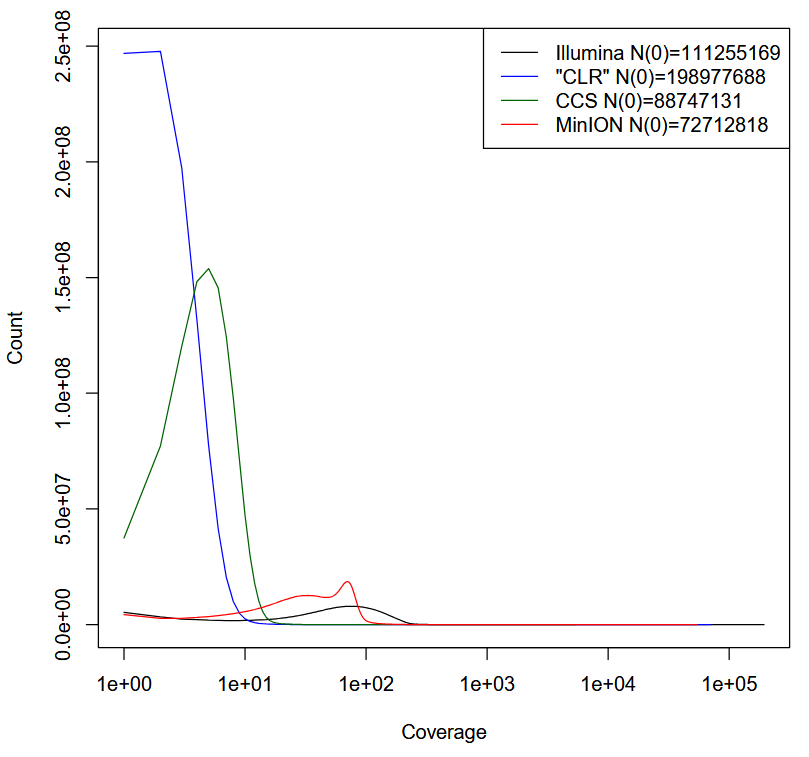


Supplementary table 1: **Genome size compilation and published *Aedes* genomes used for the assembly and annotation statistics as given in Table 1.** FCM: flow cytometric measurements. k-mer: k-mer-based estimation using Jellyfish and GenomeScope [2]. mapping: mapping-based genome size estimate based on available data types using ModEst [3]. “CLR”: constructed continuous long reads (CLR) reads. ILL: Illumina reads. CCS: circular consensus sequencing (CCS) reads, ONT: Oxford Nanopore Technology’s MinION long reads. ^X^ Total length of assembly.

| **Species** | **Assembly name** | **Assembly acc.**  **no.** | **Reference** | **Genome size [Gb]** |
| --- | --- | --- | --- | --- |
| ***Ae. j. japonicus*** | **This dataset** | | | **FCM: 1.3**  **k-mer: 0.7**  **mapping Illumina: 1.6**  **mapping “CLR”: 3.0**  **mapping CCS: 1.3**  **mapping ONT: 1.0** |
| *Ae. japonicus* | Ajap1v2 | GCA_034211315.2 | [4] | 1.4 ^x^ |
| ***Ae. koreicus*** | Akor_1.1 | GCA_024533555.2 | [5] | 1.1 ^x^ |
|  |  |  | **This study** | **FCM: 1.3** |
| *Ae. albopictus* | AalbF5 | GCF_035046485.1 | [6] | 1.3 ^x^ |
|  |  |  | [6] | FCM: 1.2 to 1.3 |
|  |  |  | [8] | 0.6 to 1.6 |
| *Ae. aegypti* | AaegL5.0 | GCF_002204515.2 | [7] | 1.3 ^x^ |
|  |  |  | [8] | 0.8 to 1.2 |


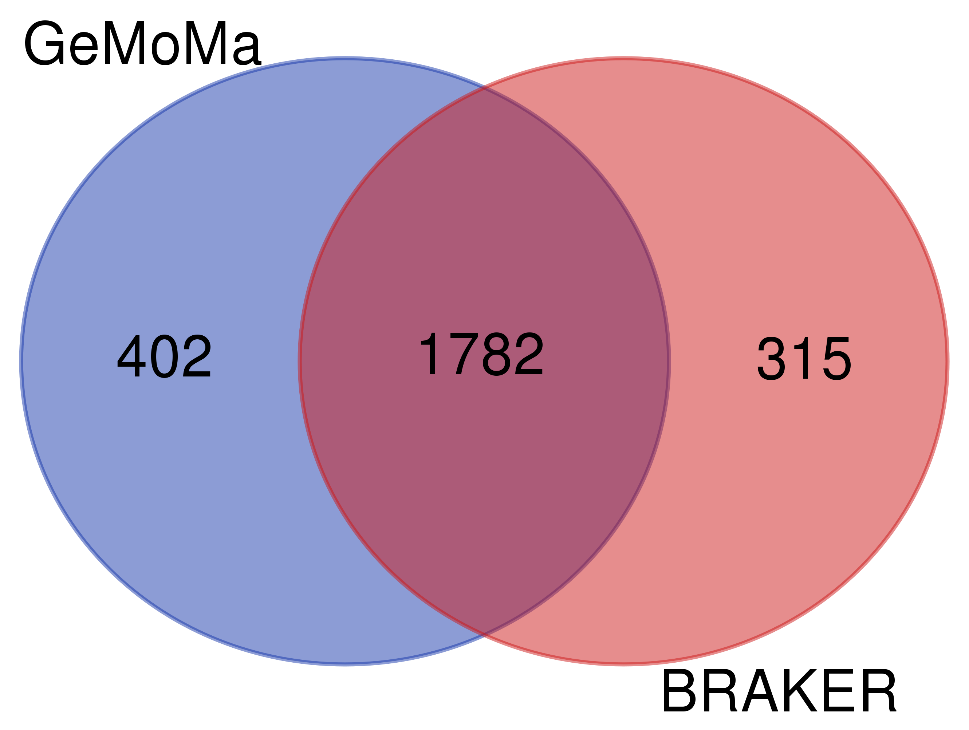


Supplementary figure 4: **Venn diagram of complete and single-copy BUSCO gene IDs of the GeMoMa** [9] **and BRAKER** [10] **annotations for *Aedes japonicus japonicus***. Both annotations show unique genes (402 and 315, respectively), which leads to the conclusion that combining both gene predictions could improve the *Aedes japonicus japonicus* annotation. The GeMoMa annotation has 2,184 complete single-copy BUSCOs and the BRAKER annotation has 2,097 complete single-copy BUSCOs. The Venn diagram was calculated and visualised with the online tool <https://bioinformatics.psb.ugent.be/webtools/Venn/>.

Supplementary table 2: **Annotation statistics of the annotation of *Aedes japonicus japonicus*.** BRAKER: De novo annotation method. GeMoMa: Reference-based annotation method. Basic contiguity (statistics calculated by a custom script by author TS) and BUSCO [11] statistics of the annotations are shown. The merged annotation was done via filtering unique BUSCO IDs and merging with GFF3toolkit.

| **Annotation statistic** | ***Ae. j. japonicus***  **BRAKER** | ***Ae. j. japonicus***  **GeMoMA** | ***Ae. japonicus***  **merged annotation** |
| --- | --- | --- | --- |
| No. genes | 23,878 | 24,644 | 33,722 |
| No. mRNA | 28,836 | 28,665 | 38,427 |
| No. CDS | 120,432 | 103,908 | 131,654 |
| Mean mRNAs/gene | 1.21 | 1.16 | 1.14 |
| Mean CDSs/mRNA | 4.18 | 3.63 | 3.43 |
| Median gene length | 2,027 | 1,902 | 1,787 |
| Median mRNA length | 2,223 | 1,948 | 1,856 |
| Median CDS length | 180 | 234 | 214 |
| Total gene space | 407,892,850 | 130,166,222 | 271,620,081 |
| Total mRNA space | 407,892,850 | 130,166,222 | 271,620,081 |
| Total CDS space | 28,457,713 | 43,817,640 | 49,788,371 |
| Single CDS mRNA | 4,854 | 5,497 | 8,912 |
| % complete BUSCOs | 91.4 | 87.1 | 92.5 |
| % single-copy BUSCOs | 62.3 | 66.5 | 69.4 |
| % duplicated BUSCOs | 29.1 | 20.6 | 23.1 |
| % fragmented BUSCOs | 2.8 | 1.9 | 2.1 |
| % missing BUSCOs | 5.8 | 11.0 | 5.4 |

Supplementary table 3: **Comparison of reference-based annotations with three (*Ae. albopictus* (GCF_035046485.1)*, Ae. aegypti* (GCF_002204515.2)*, Ae. koreicus* (GCA_024533555.2)) versus two (*Ae. albopictus* (GCF_035046485.1)*, Ae. aegypti* (GCF_002204515.2)) references.** Both reference-based annotations were done in GeMoMa. The outputs were evaluated using basic contiguity statistics (custom script by author TS) of the annotation and BUSCO [11].

| **Annotation statistics** | **Three references** | **Two references** |
| --- | --- | --- |
| No. genes | 16,707 | 24,644 |
| No. mRNA | 23,431 | 28,665 |
| No. CDS | 94,258 | 103,908 |
| Mean mRNAs/gene | 1.40 | 1.16 |
| Mean CDSs/mRNA | 4.02 | 3.63 |
| Median gene length | 1,681 | 1,902 |
| Median mRNA length | 1,745 | 1,948 |
| Median CDS length | 209 | 234 |
| Total gene space | 102,504,882 | 130,166,222 |
| Total mRNA space | 102,504,882 | 130,166,222 |
| Total CDS space | 24,019,887 | 43,817,640 |
| Single CDS mRNA | 2,177 | 5,497 |
| % complete BUSCOs | 87.5 | 87.1 |
| % single-copy BUSCOs | 61.1 | 66.5 |
| % duplicated BUSCOs | 26.4 | 20.6 |
| % fragmented BUSCOs | 2.6 | 1.9 |
| % missing BUSCOs | 9.9 | 11.0 |

| **Number of annotated protein or virus sequences (%)** | | | | | |
| --- | --- | --- | --- | --- | --- |
| **Database** | ***Ae. j. japonicus*** | ***Ae. albopictus*** | | ***Ae. aegypti*** | |
| Swiss-Prot | 28,284 (98%) | | 32,826 ( 99%) | | 28,136 ( 99%) |
| Pannzer2 DE | 14,670 (51%) | | 25,262 ( 76%) | | 24,666 ( 87%) |
| Pannzer2 GO | 14,906 (52%) | | 25,810 ( 78%) | | 25,163 ( 89%) |
| GhostKOALA KO | 12,621 (44%) | | 16,909 ( 51%) | | 17,891 ( 63%) |
| InterProScan | 23,509 (82%) | | 32,156 ( 84%) | | 27,534 ( 97%) |
| InterProScan GO | 13,265 (46%) | | 21,551 ( 65%) | | 19,396 ( 69%) |
| At least one annotation with one database | 28,458 (99%) | | 33,052 (100%) | | 28,304 (100%) |
| GO terms | 17,218 (60%) | | 27,640 ( 84%) | | 25,482 ( 90%) |
| Integrated viruses  [6] | 3,208 (60%) | | 3,731 (70%) | | 3,453 ( 65%) |

Supplementary table 4: **Functional annotations per database and per *Aedes*-species.** GO: gene ontology, DE: functional predictions with Pannzer2. KO: KEGG orthology.

Pannzer2 web version: <http://ekhidna2.biocenter.helsinki.fi/sanspanz/>. GhostKOALA web version: <https://www.kegg.jp/ghostkoala/>


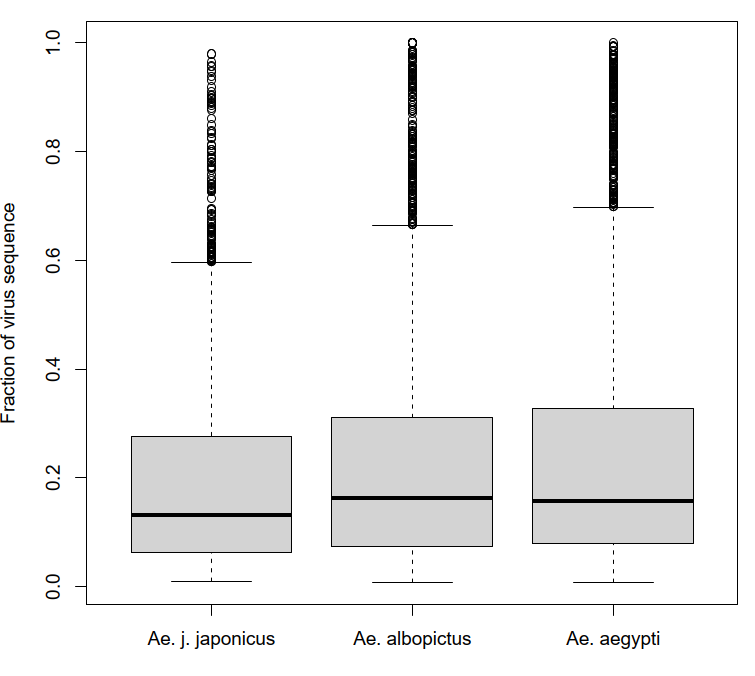


Supplementary figure 5: **Visualisation of integrated viruses** [6] **as fraction per total virus sequence length.** Shown are boxplots with median and hinges representing 25 and 75 percentiles, whiskers extend to 1,5 times the interquartile range and circles show outliers.

REFERENCES OF THE SUPPLEMENTARY MATERIAL

1. Kolmogorov M, Yuan J, Lin Y, Pevzner PA. Assembly of long, error-prone reads using repeat graphs. *Nat Biotechnol*. 2019; doi: 10.1038/s41587-019-0072-8.

2. Ranallo-Benavidez TR, Jaron KS, Schatz MC. GenomeScope 2.0 and Smudgeplot for reference-free profiling of polyploid genomes. *Nat Commun*. 2020; doi: 10.1038/s41467-020-14998-3.

3. Pfenninger M, Schönnenbeck P, Schell T. ModEst: Accurate estimation of genome size from next generation sequencing data. *Mol Ecol Resour*. 2022; doi: 10.1111/1755-0998.13570.

4. Catapano PL, Falcinelli M, Damiani C, Cappelli A, Koukouli D, Rossi P, et al. De novo genome assembly of the invasive mosquito species *Aedes japonicus* and *Aedes koreicus*. *Parasit Vectors*; doi: 10.1186/s13071-023-06048-w.

5. Nagy NA, Tóth GE, Kurucz K, Kemenesi G, Laczkó L. The updated genome of the Hungarian population of *Aedes koreicus*. *Sci Rep*. 2024; doi: 10.1038/s41598-024-58096-6.

6. Palatini U, Masri RA, Cosme LV, Koren S, Thibaud-Nissen F, Biedler JK, et al. Improved reference genome of the arboviral vector *Aedes albopictus*. *Genome Biol*. 2020; doi: 10.1186/s13059-020-02141-w.

7. Matthews BJ, Dudchenko O, Kingan SB, Koren S, Antoshechkin I, Crawford JE, et al. Improved reference genome of *Aedes aegypti* informs arbovirus vector control. *Nature*. 2018; doi: 10.1038/s41586-018-0692-z.

8. Gregory T. Animal genome size database.

9. Keilwagen J, Hartung F, Grau J. GeMoMa: Homology-Based Gene Prediction Utilizing Intron Position Conservation and RNA-seq Data. In: Kollmar M, editor. *Gene Predict*. New York, NY: Springer New York;

10. Brůna T, Hoff KJ, Lomsadze A, Stanke M, Borodovsky M. BRAKER2: automatic eukaryotic genome annotation with GeneMark-EP+ and AUGUSTUS supported by a protein database. *NAR Genomics Bioinforma*. 2021; doi: 10.1093/nargab/lqaa108.

11. Manni M, Berkeley MR, Seppey M, Simão FA, Zdobnov EM. BUSCO Update: Novel and Streamlined Workflows along with Broader and Deeper Phylogenetic Coverage for Scoring of Eukaryotic, Prokaryotic, and Viral Genomes. Kelley J, editor. *Mol Biol Evol*. 2021; doi: 10.1093/molbev/msab199.
